# Omega-3FAs Can Inhibit the Inflammation and Insulin Resistance of Adipose Tissue Caused by HHcy Induced Lipids Profile Changing in Mice

**DOI:** 10.3389/fphys.2021.628122

**Published:** 2021-02-12

**Authors:** Jing Li, Heng Zhang, Yongqiang Dong, Xian Wang, Guang Wang

**Affiliations:** ^1^Department of Endocrinology, Beijing Chao-Yang Hospital, Capital Medical University, Beijing, China; ^2^Key Laboratory of Molecular Cardiovascular Science, Department of Physiology and Pathophysiology, School of Basic Medical Sciences, Ministry of Education, Peking University, Beijing, China

**Keywords:** insulin resistance, homocysteine, adipocyte, omega-3 fatty acids, inflammation

## Abstract

The adipose Nod-like receptor protein 3 (NLRP3) inflammasome initiates insulin resistance; however, the mechanism of inflammasome activation in adipose tissue remains elusive. In this study, homocysteine (Hcy) was found to participate in insulin resistance *via* a NLRP3 inflammasome-related process. Hcy-induced activation of NLRP3 inflammasomes were observed in adipose tissue during the generation of insulin resistance *in vivo*. This animal model suggests that diets high in omega-3 fatty acids alter serum and adipose lipid profiles, and in this way, omega-3 fatty acids may reduce adipose tissue inflammation and attenuate insulin resistance.

## Introduction

Insulin resistance plays a key role in the metabolic syndrome, which includes diabetes, obesity-related atherosclerosis, and lipid disorders (Petersen and Shulman, 2018). Previously, we reported that hyperhomocysteinemia (HHcy) was an independent risk factor for development of insulin resistance (Li Y. et al., 2013). This was confirmed in studies both in humans and in a rodent model. Omega-3 fatty acids (ω-3FAs), primarily eicosapentaenoic acid (EPA, 20:5n-3) and docosahexaenoic acid (DHA, 22:6n-3), exert anti-inflammatory activities in a variety of inflammatory diseases, including obesity, diabetes, atherosclerosis, as well as other metabolic inflammatory disorders (Tortosa-Caparrós et al., 2017). Fish oil, which contains ω-3FAs, is thought to participate in lowering serum concentrations of lipids (Nicholls et al., 2018). In another study, n-3 polyunsaturated fatty acids (PUFAs) regulated hepatic genes such as peroxisome proliferators-activated receptors-alpha (PPAR-alpha) and sterol-regulatory element binding protein-1 (SREBP-1). This may modestly raise hepatic glucose production while also reducing hyperinsulinemia, but without causing peripheral insulin resistance or systemic metabolic dysfunction (Devarshi et al., 2013). In animal models, expression of such kinds of lipids biosynthesis related genes in the liver reduce hepatic steatosis (Rossmeisl et al., 2014). Nevertheless, the mechanisms by which ω-3FAs reduce adipose insulin resistance remain poorly understood.

Homocysteine (Hcy) is a sulfur-containing non-proteinogenic amino acid. Increased plasma Hcy levels [to more than 15 μM, defined as hyperhomocysteinemia (HHcy)] is an independent risk factor for cardiovascular disease and other metabolic inflammation diseases (Hainsworth et al., 2016). Patients with insulin resistance and type 2 diabetes mellitus were found to have increased plasma levels of Hcy (Li et al., 2008; Kundi et al., 2017). Treatments that lower plasma Hcy levels, including folic acid, might improve insulin sensitivity; dietary supplementation vitamin B are more effective in preventing metabolic syndrome and related vascular disease in areas where the population has a normally low folate consumption than in areas with higher dietary folate intake (Earnest et al., 2012). Our previous studies established that mice treated with oral Hcy mimic the increase of atherosclerosis and other general phenotypes in patients with HHcy (Dai et al., 2006). HHcy increases oxidative stress and its downstream signaling pathways, resulting in vascular inflammation stress and endoplasmic reticulum stress (Fu et al., 2018; Majumder et al., 2018). In a mouse model of HHcy, Hcy was enriched in adipose tissue, promoting insulin resistance and metabolic inflammation (Li et al., 2008; Li Y. et al., 2013).

We found that increased plasma levels of Hcy promotes insulin resistance by activating adipocyte and adipose tissue macrophage Nod-like receptor protein 3 inflammasomes (NLRP3). The NLRP3 inflammasome is also involved in Hcy-induced adipose insulin resistance; Hcy acted as a second signal activator of the adipocyte NLRP3 inflammasome, and the adipocyte inflammation participates in lipid disorders and insulin resistance (Zhang et al., 2018). Nevertheless, it remains unknown as to whether ω-3FA intake attenuates adipose metabolic inflammation.

Therefore, in the present study, we focused on whether ω-3FA consumption would attenuate HHcy-induced insulin resistance and tried to explain the mechanism through the histochemical changes caused by ω-3FAs.

We used ultraperformance liquid chromatography coupled to electrospray ionization quadrupole mass spectrometry (UPLC-ESI-QTOFMS)-based metabolomics analysis to measure the changes of bioactive metabolites in HHcy mice undergoing ω-3FA treatment.

## Materials and Methods

### Animals and Housing

All mice were housed under specific pathogen-free conditions in a temperature-controlled room (22°C) with a 12-h light and dark cycle and were given free access to normal chow diet (Cat. 1025, HFK Biosciences, Beijing, China) and drinking water. Wild type (WT) mice were generated from the C57BL/6J background and were obtained from Vital River Laboratories (Beijing, China). Protocols were approved by the Animal Care and Use Committee of Capital Medical University.

### HHcy Mouse Models

Mice were given water with or without DL-Hcy (1.8 g/l), and were fed a standard chow diet or a 3.3% omega-3 PUFA (33 mg/g) diet. After 6 weeks, mice were sacrificed, and the metabolic profiles of the adipose tissue were analyzed using UPLC-ESI-QTOFMS-based lipidomics. Expression levels of ceramide metabolism-related genes were measured using quantitative PCR. DL-Hcy was purchased from Sigma-Aldrich Chemicals (Cat. H4628, St. Louis, MO, United States).

### Glucose Tolerance Test and Insulin Tolerance Test

For the glucose tolerance test (GTT), mice were fasted for 12 h before the administration of glucose (1.8 g/kg, i.p.). Blood samples were drawn from a cut at the tip of the tail at 0, 30, 60, 90, and 120 min after glucose administration, and blood glucose concentrations were measured immediately. For the insulin tolerance test (ITT), mice were fasted for 4 h before the administration of insulin (1 IU/kg, i.p.). Blood samples were drawn from a cut at the tip of the tail at 0, 30, 60, 90, and 120 min after insulin administration, and blood glucose concentrations were measured immediately.

### Immunohistochemistry

F4/80 (Cat.ab16911, Abcam, Abcam Cambridge, United Kingdom) expression in adipose tissue was examined by immunohistochemistry using 7-μm sections of the eWAT. The sections were blocked with 5% bovine serum albumin (BSA) for 1 h and incubated overnight at 4°C with F4/80 antibody (1:500). After washing, the sections were incubated with the Horseradish Peroxidase (HRP)-conjugated anti-rabbit IgG (Cat. sc-2004, Santa Cruz, Dallas, TX, United States) secondary antibody (1:1000) for 1 h. The DAB method was used to detect the F4/80 signal.

### Lipidomics Analysis

The serum and adipose sample preparations and the lipidomics analyses were undertaken as described previously (Jiang et al., 2015). In brief, epididymal white adipose tissue (eWAT) (20 mg) were homogenized with ultrapure water (200 μl) and then extracted with chloroform-methanol (2:1) solution (1,000 μl). The samples were incubated at 37°C for 30 min and subsequently centrifuged at 16,000 *g* for 20 min at 4°C. The lower organic phase (approximately 500 μl) was collected and evaporated. The organic residue was dissolved in isopropanol-acetonitrile (1:1) solution (100 μl). Samples were analyzed using the Thermo Scientific Dionex UltiMate 3000 Rapid Separation LC system (Thermo Fisher Scientific, Waltham, MA, United States). Peak extraction and integration were performed using Xcalibur 2.2 SP1.48 software (Thermo Fisher Scientific, Waltham, MA, United States).

### Western Blot

Total protein was isolated with RIPA lysis buffer (Cat. P0013C, Beyotime Biotechnology, Shanghai, China). Total protein was subjected to sodium dodecylsulfate polyacrylamide gel electrophoresis on 10 or 12% running gels and then transferred to polyvinylidene fluoride membranes. The membranes were incubated with 10% BSA in Tris Tween-buffered saline at room temperature for 1 h, with various primary antibodies at 4°C for 12 h and with a HRP-conjugated secondary antibody for 1.5 h. The bands were exposed using the ChemiDOC XRS System (Bio-Rad, Hercules, CA, United States). Anti-NLRP3 (Cat. AF-401-NA) antibody was purchased from R&D Systems (Minneapolis, MN, United States). Anti-β-actin (Cat. 8457) antibody was purchased from Cell Signaling Technology (Danvers, MA, United States). The HRP-conjugated anti-rabbit IgG (Cat. sc-2004), HRP-conjugated anti-goat IgG (Cat. sc-2020), and HRP-conjugated anti-mouse IgG (Cat. sc-2005) secondary antibodies were all purchased from Santa Cruz Biotechnology (Dallas, TX, United States).

### Quantitative PCR Measurement of mRNA Levels

Total RNA was isolated using TRIzol Reagent (Cat. 15596018, Thermo Fisher Scientific, Waltham, MA, United States). Total RNA (2 μg) was reverse transcribed using 5X All-In-One RT MasterMix (Cat. G490, abm, Richmond, BC, Canada). Quantitative PCR (qPCR) analysis was performed using RealStar Green Power Mixture (Cat. A314, GenStar, Beijing, China) and run on an Mx3000 Multiplex Quantitative PCR System (Agilent, La Jolla, CA, United States). The amount of the PCR products formed in each cycle was evaluated using the fluorescence of SYBR Green I. The results were analyzed using Stratagene Mx3000 software. The qPCR primer sequences are shown in Table 1.

**TABLE 1:** The qPCR primer sequences.

Mus musculus hypoxia inducible factor 1, alpha subunit (Hif1a). NM_001313919.1	Forward primer AGGATGAGTTCTGAACGTCGAAA Reverse primer CTGTCTAGACCACCGGCATC
Mus musculus phospholipase A2, group XVI. BC024581.1	Forward primer CGCGTGGGCGAGGAG Reverse primer CTTCACTTGGAGGAGCCAGG
Mus musculus solute carrier family 2 (facilitated glucose transporter), member 1 (Slc2a1). NM_011400.3	Forward primer CGATCTGAGCTACGGGGTCT Reverse primer AGAACTCCTCAATAACCTTCTGGG
Mus musculus pyruvate dehydrogenase kinase, isoenzyme 1 (Pdk1). NM_172665.5	Forward primer CATACAGCCGCAGGTTGGC Reverse primer AGCATTCACTGACCCGAAGT
Mus musculus actin, beta (Actb), NM_007393.5	Forward primer GCCTTCCTTCTTGGGTATGGAA Reverse primer CAGCTCAGTAACAGTCCGCC

### Statistical Analysis

The data were expressed as means ± SD and were analyzed using GraphPad Prism (GraphPad Software, La Jolla, CA, United States). For metabolomics analysis, the data were analyzed using MetaboAnalyst 3.0. One-way ANOVA with Tukey’s multiple comparisons test (between multiple groups) and unpaired Student’s t test (between two groups) were used as appropriate. *P* < 0.05 was considered significant.

## Results

### ω-3FAs Treatment Improves Hcy-Induced Insulin Resistance in a Mouse Model

**FIGURE 1:**
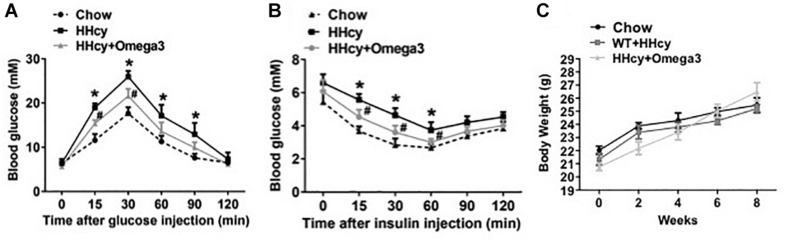
ω-3FAs treatment improves Hcy-induced insulin resistance in a mouse model. **(A)** Plasma glucose levels of Intraperitoneal glucose tolerance test (IPGTT) in a mouse model (*n* = 7). **(B)** Plasma glucose levels of insulin tolerance test (ITT) in a mouse model (*n* = 7). **(C)** Body weight in the mouse model (*n* = 7). All data are presented as the means ± SD. *P < 0.05 vs. control group, #*P* < 0.05 vs. HHcy group; mice given Hcy (1.8 g/l), ω-3FAs (3.3%) in the drinking water for 6 weeks.

To investigate the precise mechanisms underlying the effects fish oil on Hcy-induced insulin resistance, WT mice were given Hcy with or without fish oil in the drinking water (Hcy 1.8 g/l, ω-3FAs 3.3%, 6 weeks). IPGTT and ITT revealed marked glucose intolerance and insulin resistance in the Hcy-treated mice (Figures 1A,B), while the body weights were not significantly different from those of controls after fish oil treatment (Figure 1C).

### ω-3FAs Intake Decreases the Production of Sphingolipids and Free Fatty Acids in Serum and Changes the Lipid Profile of Adipose Tissue Under Normal Diet

**FIGURE 2:**
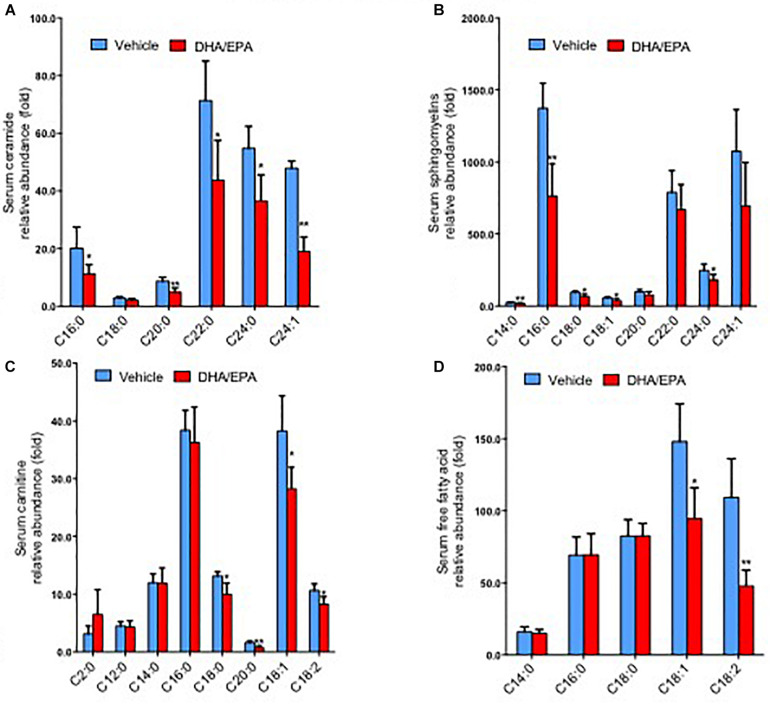
ω-3FAs intake decreases the production of sphingolipids and free fatty acids in serum and adipose under normal diet. **(A)** Lipidomics analysis of the abundance of serum ceramide under normal diet. **(B)** Lipidomics analysis of the abundance of serum sphingomyelins under normal diet. **(C)** Lipidomics analysis of the abundance of serum carnitine under normal diet. **(D)** Lipidomics analysis of the abundance of serum free fatty acids under normal diet. *P < 0.05 vs control group, **P < 0.01 vs control group, *n* = 7.

**FIGURE 3:**
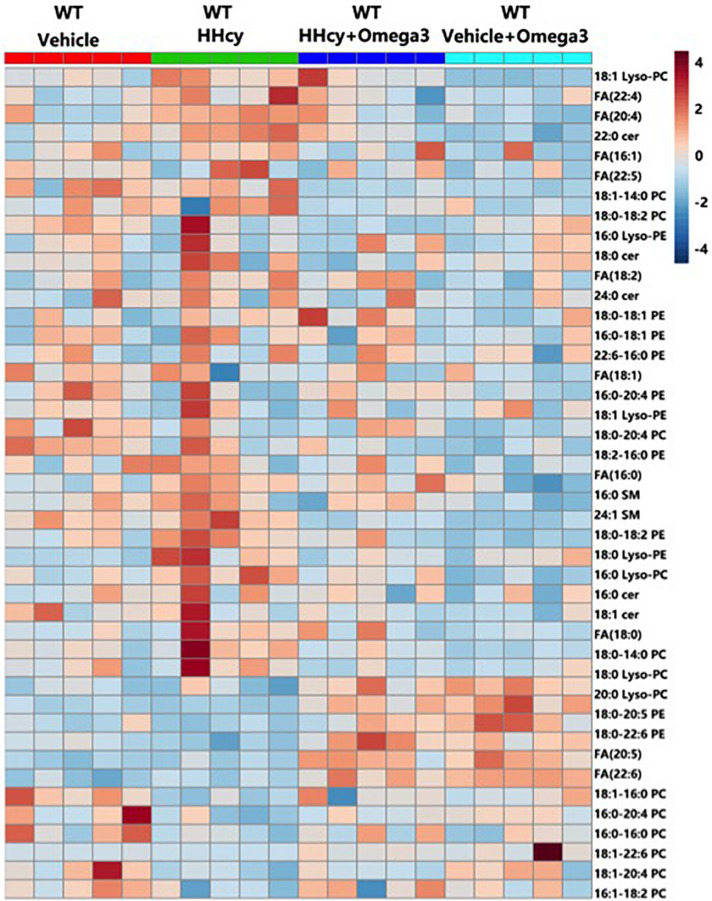
Heatmap of phospholipids and FAs in the adipose tissue of Hcy-treated mice. Seven-week-old WT mice were fed normal chow diet and were given Hcy (1.8 g/L) or vehicle in the drinking water with or without ω-3FA (3.3%) for 6 weeks.

To further characterize the exact lipid profile changes because of ω-3FAs treatment, a non-targeted lipidomics assay was performed in serum. ω-3FAs intake decreased the production of sphingolipids and free fatty acids in fat tissue under normal diet (*n* = 7 per group) (Figures 2A–D). A heatmap of phospholipids and FAs revealed increased lyso-PC and PUFA levels in the adipose tissue of Hcy-treated mice that represents a lipid metabolic pattern distinct from that of vehicle-treated mice after ω-3FAs intake (Figure 3).

### ω-3FAs Supplementation Significantly Changes the Lipid Profile in Adipose Tissue

**FIGURE 4:**
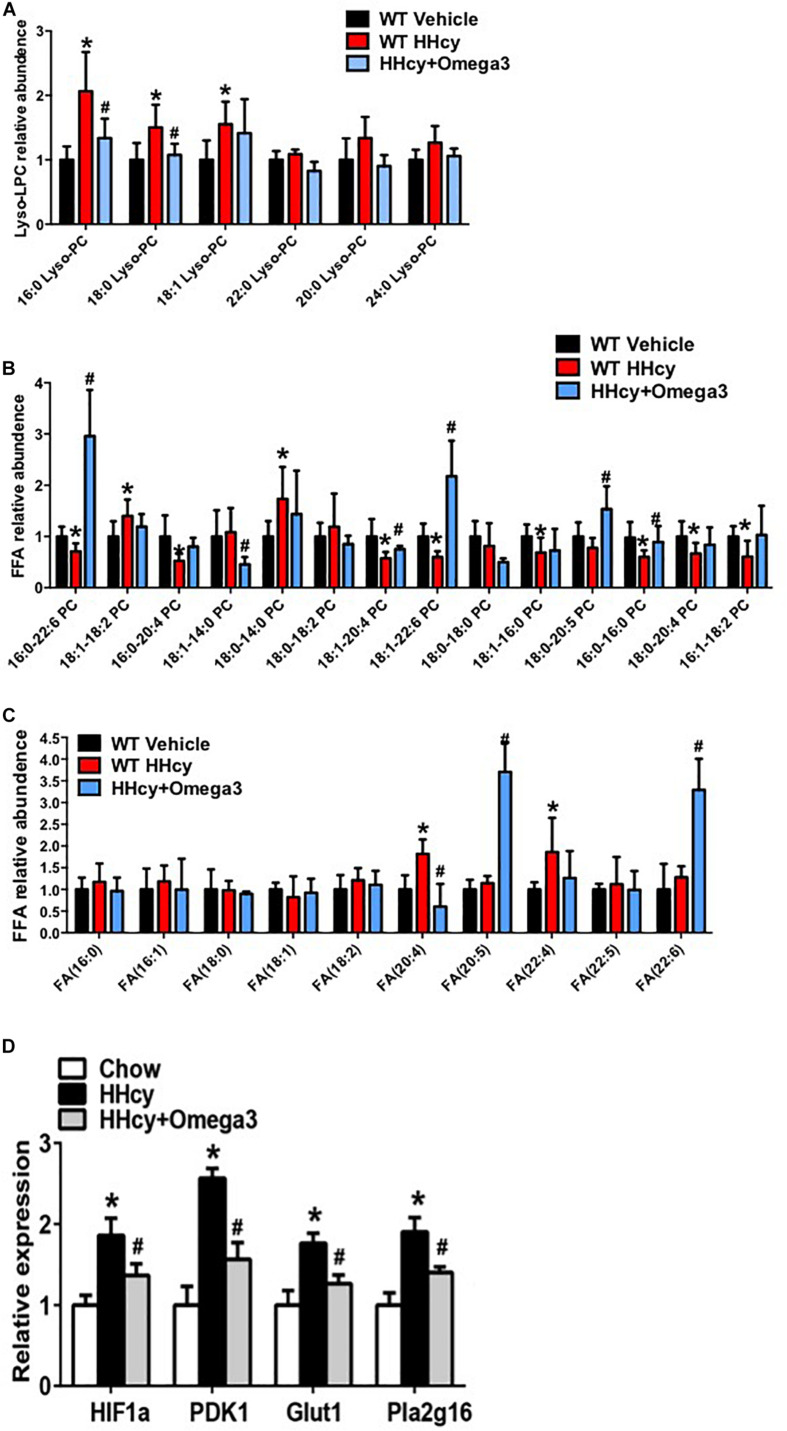
ω-3FAs supplementation significantly changes the lipid profile of adipose tissue. **(A)** Lipidomics analysis of the lyso-PC profile of mouse adipose tissue; (*n* = 6 per group). **(B)** Lipidomics analysis of the PC profile of mouse adipose tissue; (*n* = 6 per group). **(C)** Lipidomics analysis of the FA profile of mouse adipose tissue; (*n* = 6 per group). Seven-week-old WT mice were fed normal chow diet and were given Hcy (1.8 g/L) or vehicle in the drinking water with or without ω-3FAs (3.3%) for 6 weeks. Two-tailed Student’s *t*-test: *P < 0.05 vs. control group, #*P* < 0.05 vs. HHcy group. **(D)** qPCR analysis of mRNA levels of HIF-1, PDK1, Glut1, and Pla2g16 in mouse adipose tissue; (*n* = 6 per group).

Levels of lyso-PC (16:0), lyso-PC (18:0), lyso-PC (18:1), PC (16:0-22:6), PC (18:1-14:0), PC (18:1-20:4), PC (18:1-22:6), PC (18:0-20:5), PC (16:0-20:0), FA (20:4), and FA (22:4) were altered by Hcy treatment while tending to remain normal after ω-3FA intake (Figures 4A–C). The generation of lyso-PC and PUFA depends on phospholipase A2 (PLA2), which hydrolyzes the *sn*-2 position of phosphatidylcholine (PC) (Murakami et al., 2011). In agreement with the lipidomics data, PLA2 activity was elevated in the adipose tissue of Hcy-treated mice (Figure 4D).

### ω-3FAs Attenuate Hcy-Induced NLRP3 Inflammasome Activation in Adipose Tissue

**FIGURE 5:**
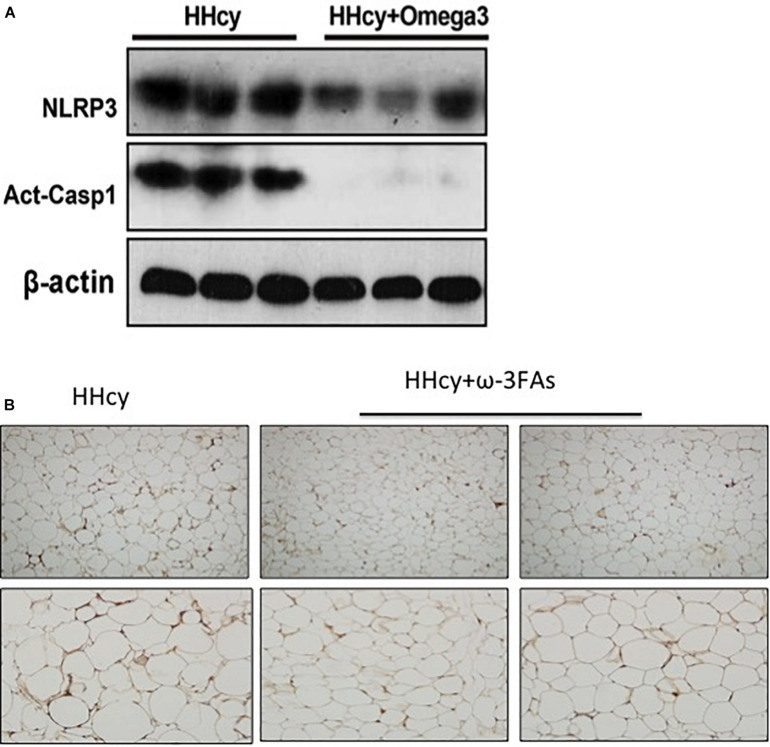
Activation of the Hcy-induced NLRP3 inflammasome was attenuated by ω-3FAs in adipose tissue. **(A)** Western blot analysis of Act-CASP1 and NLRP3 protein levels in adipose tissue; **(B)** Immunohistochemical study of inflammation related molecule F4/80 in mouse adipose tissue, (*n* = 6 per group). Seven-week-old WT mice were fed normal chow diet and were given Hcy (1.8 g/L) or vehicle in the drinking water with or without ω-3FAs (3.3%) for 6 weeks. Two-tailed Student’s *t*-test: *P < 0.05 vs. control group, #*P* < 0.05 vs. HHcy group, *n* = 7.

Homocysteine was previously shown to increase the HIF1α protein levels in podocytes (Li C. et al., 2013). Increased HIF1α levels, and the glycolysis-associated genes, PDK1 and Glut1 mRNA level were also observed in the adipose tissue of Hcy-treated mice (Figure 4D). PLA2G16 is a PLA2 and is specifically expressed in adipocytes with a preference toward hydrolysis of PC (Duncan et al., 2008). Expression levels of Pla2g16 were downregulated after ω**-**3FA intake that was highly expressed in Hcy-induced insulin resistance mice while levels of PDK1 and Glut1 decreased (Figure 4D). Protein levels of NLRP3 and Act-Casp1 were measured using western blotting. After Hcy treatment, NLRP3 levels were elevated in adipose tissue, and ω-3FAs inhibited levels of Hcy-up-regulated inflammatory markers (Figure 5A). In order to verify that fish oil treatment can improve the inflammation in adipose tissue, we carried out the immunohistochemistry of adipose tissue. The results showed that after 6 weeks of ω-3FAs treatment, the expression of F4/80 in adipose tissue of mice decreased, indicating that the inflammation of adipose tissue was alleviated (Figure 5B).

## Discussion

Clinical trials assessing effect of ω**-**3FAs in primary and secondary prevention of cardiovascular disease have produced conflicting findings. Fish oil rich in ω-3FAs supplements have been widely used and recommended for prevention and treatment of lipid disorders and metabolic disease, and the clinical utility of ω-3FAs in lowering triglyceride levels is well known (Mansoori et al., 2015). There is also evidence suggesting that the Mediterranean diet supports addition of fish to the diet for prevention of cardiovascular disease (Guasch-Ferré et al., 2017). Mechanisms by which ω**-**3FAs exert their anti-inflammatory effects are not fully understood.

Homocysteine and C2-ceramide cause murine cerebral endothelial cells metabolic disorder by activating the Asm-ceramide pathway (Lee et al., 2013). Oxidative stress and *de novo* ceramide synthesis could induce NLRP3-inflammasome activation. It was found that ω**-**3FAs reduced nuclear factor kappa B-mediated inflammation in the liver through effects on inflammasome signaling (Feng et al., 2018). One study in 3T3-L1 adipocytes suggested that inhibition of IL-18, IL-1β, and caspase-1 by DHA and EPA were predominantly dependent upon adiponectin (Oster et al., 2010). These findings suggest that Hcy might induce inflammation and ω-3FAs might attenuate the inflammation as well as insulin resistance as shown in the present study.

Because the NLRP3 inflammasome is activated in human mesenchymal stem cells *via* lipopolysaccharide and palmitic acid, and plasma (Fu et al., 2019) levels are increased in the context of obesity (Wang et al., 2017), Hcy might be one of the mediators involved in obesity-induced insulin resistance. Oral intake of ω**-**3FAs as a treatment could turn over HHcy-induced lipid profile alterations in adipose tissue, and may modulate inflammation factors that have potential therapeutic functions in reducing adipose tissue inflammation-induced insulin resistance.

We found that the Hcy induced alterations in adipose tissue lipid profiles, confirming findings of our previous study. The NLRP3 inflammasome is activated by HHcy treatment and promotes insulin resistance and adipose inflammation. Increased expression levels of Pla2g16 mRNA as a marker of inflammatory activation are attenuated after ω-3FAs intake, hinting that adipose insulin resistance might be reduced secondary to the lipid profile modifications, especially in adipose tissue.

C12-16 saturated and monounsaturated fatty acids, ceramide, and lyso-PC have been reported to act as second signal activators of the NLRP3 inflammasome (Matsuzaka et al., 2012; Scheiblich et al., 2017; Zhang et al., 2018). Extracellular lyso-PC has been reported to activate G protein-coupled receptors (GPRs), GPR132 and GPR4, mediating its functions (Meyer zu Heringdorf and Jakobs, 2007) and participating in lyso-PC-induced Ca^2+^ influx and immune cell inflammation (Kabarowski, 2009; Khan et al., 2010), which may be involved in lyso-PC-induced NLRP3 inflammasome activation.

In the present study, lyso-PC was found to activate adipose NLRP3 inflammasomes. Lyso-PC also acts as a lipid mediator in response to inflammation (Kabarowski, 2009). The contrasting effects of lyso-PC on insulin resistance and adipose tissue inflammation may be due to the different species of lyso-PC. These findings support our finding that Hcy might induce inflammation in a lipid profile-affecting manner, and the alteration in the inflammation-inducing lipid profile might be affected by ω-3FAs administration.

Previous studies showed that adipose tissue insulin resistance is often accompanied by activation of inflammasomes due to hypoxia. PLA2G16 was first recognized as a type II tumor suppressor gene (Sers et al., 1997) and was identified as a PPARγ target gene, expressed specifically in adipose tissue (Hummasti et al., 2008; Uyama et al., 2009). Knockout of *Pla2g16* induced insulin resistance in mice (Jaworski et al., 2009), a finding that appears inconsistent with the present results. In our study, expression levels of HIF-1α, PLA2G16, PDK1, and Glut1 mRNA were upregulated by Hcy treatment, suggesting that Hcy induces inflammation-related insulin resistance in adipocytes, while the abnormally elevated levels of inflammation and insulin resistance were relieved after supplementation with ω-3FAs.

We acknowledge that there are limitations to our studies, including the lack of mechanistic data. Our study focused on adipose and may have reflected mixed contributions from cell types populating adipose tissue (i.e., adipocytes, adipose immune cells, adipose progenitor cells, and vasculature). Finally, in this short-term study, prediction of long-term effects and outcomes was not feasible. Despite these limitations, our study adds to the emerging body of knowledge regarding potential beneficial effects of fish oils and their derivatives.

In summary, this study demonstrated that Hcy activates the NLRP3 inflammasomes in adipocytes in a lyso-PC-dependent manner while HHcy induces insulin resistance in adipose tissue. Addition of ω-3FAs in the diet may modulate inflammatory processes.

## Data Availability Statement

The raw data supporting the conclusions of this article will be made available by the authors, without undue reservation.

## Ethics Statement

The animal study was reviewed and approved by Animal Care and Use Committee of Capital Medical University.

## Author Contributions

JL designed, performed, analyzed, and interpreted the majority of animal and biochemical experiments, and drafted the manuscript. HZ and YD supported the animal experiments, and performed and analyzed the lipidomics analysis. XW and GW designed, planned, and interpreted the study. All authors contributed to the article and approved the submitted version.

## Conflict of Interest

The authors declare that the research was conducted in the absence of any commercial or financial relationships that could be construed as a potential conflict of interest.
